# CRISPR/Cas9 Genome Editing for Tissue‐Specific In Vivo Targeting: Nanomaterials and Translational Perspective

**DOI:** 10.1002/advs.202305072

**Published:** 2023-09-05

**Authors:** Deepak Kumar Sahel, Lalitkumar K. Vora, Aishwarya Saraswat, Saurabh Sharma, Jasmin Monpara, Anisha A. D'Souza, Deepakkumar Mishra, Kamatham Pushpa Tryphena, Satoru Kawakita, Shahid Khan, Mohd Azhar, Dharmendra Kumar Khatri, Ketan Patel, Raghu Raj Singh Thakur

*Adv. Sci*. **2023**, *10*, 2207512

DOI: 10.1002/advs.202207512

In the original published article, Satoru Kawakita and Shahid Khan affiliations are swapped. Please find below the correct affiliations.

D. K. Sahel

Department of Pharmacy

Birla Institute of Technology and Science‐Pilani

BITS‐Pilani, Vidya Vihar, Pilani, Rajasthan 333031, India

L. K. Vora, D. Mishra, R. R. Singh Thakur

School of Pharmacy

Queen's University Belfast

97 Lisburn Road, Belfast BT9 7BL, UK

E‐mail: L.Vora@qub.ac.uk

A. Saraswat, K. Patel

College of Pharmacy & Health Sciences

St. John's University

Queens, NY11439, USA

S. Sharma, S. Kawakita

Terasaki Institute for Biomedical Innovation

Los Angeles, CA 90064, USA

J. Monpara

Department of Pharmaceutical Sciences

University of Sciences

Philadelphia, PA 19104, USA

A. A. D'Souza

Graduate School of Pharmaceutical Sciences and School of Pharmacy

Duquesne University

Pittsburgh, PA 15282, USA

K. P. Tryphena, D. K. Khatri

Molecular and Cellular Neuroscience Lab

Department of Pharmacology and Toxicology

National Institute of Pharmaceutical Education and Research

(NIPER)‐Hyderabad

Telangana 500037, India

E‐mail: dharmendra.niperhyd@nic.in

S. Khan

Department of Biomedical Engineering

University of California

Davis, CA 95616, USA

M. Azhar

Research and Development Tata Medical and Diagnostics Limited

Mumbai, Maharashtra 400001, India

